# Diabetes and Cataracts Development—Characteristics, Subtypes and Predictive Modeling Using Machine Learning in Romanian Patients: A Cross-Sectional Study

**DOI:** 10.3390/medicina61010029

**Published:** 2024-12-28

**Authors:** Adriana Ivanescu, Simona Popescu, Adina Braha, Bogdan Timar, Teodora Sorescu, Sandra Lazar, Romulus Timar, Laura Gaita

**Affiliations:** 1 Doctoral School of Medicine, “Victor Babes” University of Medicine and Pharmacy, 300041 Timisoara, Romania; adriana.ivanescu@umft.ro (A.I.); sandra.lazar@umft.ro (S.L.); 2 Second Department of Internal Medicine, “Victor Babes” University of Medicine and Pharmacy, 300041 Timisoara, Romania; braha.adina@umft.ro (A.B.); bogdan.timar@umft.ro (B.T.); sorescu.teodora@umft.ro (T.S.); timar.romulus@umft.ro (R.T.); gaita.laura@umft.ro (L.G.); 3 Opticlass Ophtalmology Clinic, 300012 Timisoara, Romania; 4 Department of Diabetes, “Pius Brînzeu” Emergency County Hospital, 300723 Timisoara, Romania; 5 Centre for Molecular Research in Nephrology and Vascular Disease, “Victor Babes” University of Medicine and Pharmacy, 300041 Timisoara, Romania; 6 First Department of Internal Medicine, “Victor Babes” University of Medicine and Pharmacy, 300041 Timisoara, Romania; 7 Department of Hematology, Emergency Municipal Hospital, 300254 Timisoara, Romania

**Keywords:** diabetes, cataracts, cataracts subtype, age-related cataracts, machine learning prediction

## Abstract

*Background and Objectives:* Diabetes has become a global epidemic, contributing to significant health challenges due to its complications. Among these, diabetes can affect sight through various mechanisms, emphasizing the importance of early identification and management of vision-threatening conditions in diabetic patients. Changes in the crystalline lens caused by diabetes may lead to temporary and permanent visual impairment. Since individuals with diabetes are at an increased risk of developing cataracts, which significantly affects their quality of life, this study aims to identify the most common cataract subtypes in diabetic patients, highlighting the need for proactive screening and early intervention. *Materials and Methods:* This study included 201 participants with cataracts (47.6% women and 52.4% men), of whom 105 also had diabetes. With the use of machine learning, the patients were assessed and categorized as having one of the three main types of cataracts: cortical (CC), nuclear (NS), and posterior subcapsular (PSC). A Random Forest Classification algorithm was employed to predict the incidence of different associations of cataracts (1, 2, or 3 types). *Results:* Cataracts have been encountered more frequently and at a younger age in patients with diabetes. CC was significantly more frequent among patients with diabetes (*p* < 0.0001), while the NS and PSC were only marginally, without statistical significance. Machine learning could also contribute to an early diagnosis of cataracts, with the presence of diabetes, duration of diabetes, or diabetic polyneuropathy (PND) having the highest importance for a successful classification. *Conclusions:* These findings suggest that diabetes may impact the type of cataract that develops, with CC being notably more prevalent in diabetic patients. This has important implications for screening and management strategies for cataract formation in diabetic populations.

## 1. Introduction

Diabetes is a chronic metabolic disorder that has seen a rapid rise in prevalence across both developed and developing countries, with projections indicating it may soon reach epidemic levels. Globally, diabetes affects approximately 537 million adults aged 20–79 years as of 2021, with projections suggesting this number could rise to 643 million by 2030 and 783 million by 2045 [1,2]. This chronic disease poses a significant economic burden on the healthcare system worldwide. According to the American Diabetes Association, the cost attributable to diabetes had a 7% increase from 2017 to 2022, reaching an average annual cost of $19,736 and a total estimated cost of $412.9 billion only in the US [3]. In Romania, the prevalence of diabetes is estimated at around 11% among adults, with an increasing trend due to lifestyle changes and aging populations. The economic burden associated with diabetes is substantial, with costs attributed to direct medical expenses and indirect costs related to lost productivity estimated at billions annually [4]. Diabetes represents a serious health challenge, contributing to significant morbidity through microvascular complications (neuropathy, chronic kidney disease, or retinopathy), as well as macrovascular issues like ischemic heart disease and peripheral vascular disease. Beyond diabetic retinopathy, diabetes is associated with multiple other ocular complications, including cataracts, diabetic papillopathy, and glaucoma [5,6].

Diabetes can affect the eyes through various mechanisms, highlighting the importance of identifying potential causes of vision-threatening conditions in these patients. Hyperglycemia also induces changes to the crystalline that can lead to both temporary and lasting visual impairment [7,8].

The link between diabetes and cataract development is well-established in the literature [9]. Risk factors for cataract development in individuals with diabetes include poor metabolic control, elevated HbA1c levels, potential genetic predisposition, and glucocorticoid treatments. Studies indicate that patients with diabetes are up to five times more likely to develop cataracts, often at a younger age than those without this condition [8].

The opacification of the eye characterizes cataracts’ normally transparent lens, ranging from partial to complete. This condition reduces the lens’ clarity, leading to impaired visual acuity. It can also obstruct the ophthalmologist’s view of the eye’s posterior segment and make certain treatments, such as retinal photocoagulation, impossible [10,11]. Cataracts are classified into three primary types: cortical (CC), nuclear sclerosis (NS), and posterior subcapsular (PSC). The progression of cataracts varies depending on the type and factors, such as an individual’s overall health and environmental influences [12,13]. The effect of cataracts on vision depends on the location of the lens opacity, resulting in distinct symptoms for each type. Nuclear cataracts, with opacities concentrated at the center of the lens, tend to affect distal vision more than proximal vision.

In contrast, cortical cataracts, characterized by spoke-like opacities, often cause glare and monocular diplopia [13]. When the opacity is located just behind the posterior lens capsule (PSC), it can cause significant visual impairment. The severity of symptoms, including decreased contrast sensitivity or visual acuity, glare, or impaired color vision, can vary depending on the stage of cataracts [14]. Each subtype of cataracts can develop independently, but they are more commonly found to occur together [8]. In patients with diabetes, current literature shows a higher prevalence of PSC and CC cataract subtypes [15], while NS is more commonly observed in patients without diabetes [16].

Cataract is the leading cause of vision loss worldwide. The proportion of blindness caused by cataracts varies significantly across regions, accounting for 5% of blindness in developed countries, while in poorer or remote areas, it can represent 50% or more of all cases of vision loss [17]. Cataracts are known to occur 2–5 times more frequently in patients with diabetes, and this frequency can increase to 15–25 times in individuals with diabetes who are under 40 years of age [18]. Cataracts, in combination with diabetes, represent a significant burden regarding both health and the economy, particularly in developing countries where diabetes management is insufficient and cataract surgery is often out of reach. Healthcare providers, researchers, and academics should recognize cataracts in diabetic patients as a critical and specialized issue. However, despite the significance of this condition, there is a lack of local literature addressing the prevalence and risk factors associated with the development and progression of cataracts in individuals with diabetes [11,19,20].

The success of treatment outcomes varies for each patient, with criteria differing based on individual needs, lifestyle, and medical conditions. Successful outcomes may include reduced visual symptoms, improved visual function, achievement of the desired refractive state, and enhanced mental well-being [21,22].

Given that patients with diabetes are at a higher risk of developing cataracts, which significantly impact their quality of life, this study aims to evaluate the most common cataract subtypes in diabetic patients and the potential benefits of including machine learning for its early diagnosis while raising awareness among physicians about the importance of screening and early detection in this population.

## 2. Materials and Methods

### 2.1. Study Design and Population

This cross-sectional, non-interventional research was conducted at the Emergency County Hospital “Pius Brinzeu” Timisoara between 16 July 2024 and 16 September 2024.

In this study, we have enrolled a final number of 201 participants (103 females, 98 males) from a total of 482 adult patients (over the age of 18) who attended their prescheduled visits in the polyclinic, while 103 declined to participate and 178 did not meet the study’s eligibility criteria. Exclusion criteria referred to neurological or psychiatric disorders as well as severe cognitive impairment that could prevent patients from providing informed consent. Institutionalized patients or those who presented medical pathologies that required hospitalization for the duration of the study were also excluded. In addition, patients with significant ocular surface pathologies or important corneal opacities impeding an adequate anterior segment evaluation were excluded. Informed consent was obtained from all participants, none being involved in this study’s development. The present research was conducted according to the Declaration of Helsinki (2013 version) and was approved by the Ethics Committee of the Emergency County Hospital “Pius Brinzeu” Timisoara (approval no. 473/15 July 2024).

The patients who were included underwent the general standardized evaluation performed by their physician and a complete ocular examination performed by a trained ophthalmologist. The ophthalmologic evaluation was centered on identifying cataracts and cataract subtypes, although it also included a screening of the most common ophthalmologic conditions, and it reported the results for the right and left eye to reflect the method, not specifically the differences between them.

Anthropometric and demographic data were retrieved from all participants’ medical charts, while the smoker status was self-reported.

Study participants were divided into two subgroups depending on the presence or absence of diabetes diagnosis. In the diabetes group, research protocol included, besides a priorly established diabetes diagnosis, the assessment of disease duration and metabolic control reflected by the current and maximum hemoglobin A1c (HbA1c) levels.

### 2.2. Data Collection and Medical Assessment

Demographic data, such as gender and age, and anthropometric data, such as weight and height, were identified from each patient’s medical records. Body mass index (BMI) was calculated using the metric system as weight (kg)/height^2^ (m). Diabetes diagnosis has been established according to one or more of the following criteria: fasting plasma glucose levels greater than 7.0 mmol/L (126 mg/dL), a 2-h post-load plasma glucose level above 11.1 mmol/L (200 mg/dL), or an HbA1c level above 6.5% (48 mmol/mol). The interdisciplinary diagnoses of hypertension (HTN), cardiovascular disease (CVD), chronic kidney disease (CKD), or liver disease have been established through interdisciplinary consults of cardiology, gastroenterology, and nephrology. In contrast, the presence of diabetic neuropathy was established using the pinprick, 10 g monofilament, temperature, and vibration test.

A comprehensive slit lamp biomicroscope (Topcon SL-D2 biomicroscope, Tokyo, Japan) examination with prior pupil dilation was conducted on all participants by a trained ophthalmologist. Cataract diagnosis was formulated by evaluating the presence and type of lens opacities, focusing on the three main cataract subtypes—CC, NS, and PSC cataract, according to the Lens Opacity Classification System (LOCS) III. CC was diagnosed in the presence of wedge-shaped or spoke-like opacities in the periphery of the crystalline lens. PSC was considered when granular opacities or opacified plagues were identified in the posterior cortex, while NS cataract was diagnosed in the presence of a hardened, yellow lens nucleus. Intraocular pressure (IOP) was also measured for all patients after applying anesthetic drops and fluorescein dye using a Perkins handheld applanation tonometer (Perkins Mk3 tonometer, Haag- Streit, Koniz, Switzerland).

### 2.3. Statistical Analysis

Statistical analysis was conducted using the MedCalc^®^ Statistical Software version 23.0.5 (MedCalc Software Ltd., Ostend, Belgium; https://www.medcalc.org; accessed on 30 August 2024) and JASP version 0.19.0 (University of Amsterdam, The Netherlands; https://jasp-stats.org; 30 August 2024) [23].

Data normality was assessed with the Shapiro–Wilk test. Due to the non-Gaussian distribution of the data, numerical variables are presented as medians with the 25th and 75th interquartile ranges (IQR), and group comparisons were made using the Mann–Whitney U test. Categorical variables were expressed as frequencies and percentages, with group comparisons carried out via the Chi-squared test or Fisher’s exact test, as appropriate. Descriptive statistics, including percentages, medians, and IQR, were used to summarize the characteristics of study groups compared to factors like gender and the presence of diabetes. A two-tailed *p*-value below 0.05 was considered statistically significant.

To predict the incidence of different associations of cataracts (1, 2, or 3 types), a Random Forest Classification algorithm was employed to handle complex interactions among a large set of predictor variables: diabetes, age, sex, weight, height, BMI, nutritional status–obesity (OB), smoking, HTN, dyslipidemia, CVD, CKD, liver disease, PND. The model was optimized with respect to the out-of-bag accuracy. The dataset was split into training and testing subsets to validate the predictive model’s performance and ensure that the model generalizes well to unseen data. We calculated accuracy, sensitivity, and area under the Receiver Operating Characteristic (ROC) curve to evaluate the predictive model’s performance with true positive and negative rates across different threshold settings. An AUC value above 0.5 indicated discriminative power. To identify which predictors significantly contribute to predicting cataract associations, we provided the variable importance with mean decrease in accuracy, total increase in node purity, and mean dropout loss. A higher mean decrease in accuracy for a variable suggests that it plays a critical role in the model’s predictions. A higher total increase in node purity for a specific variable suggests that it effectively separates different cataract association classes within the model. A higher mean dropout loss indicates that removing this variable leads to a significant decline in model performance.

## 3. Results

### General Characteristics of the Studied Lot

A total number of 201 patients were enrolled in the present study, of which 51.2% (103/201) were women and 48.8% (98/201) were men, with a median age of 72 years, median weight of 80 kg, and a median BMI of 27.3 kg/m^2^. In the study group, women were older than men (74.0 years compared to 69.0 years, *p* < 0.0001). Men were significantly taller and had higher weights than women. However, BMI was similar in both men and women (*p* = 0.7). The median IOP was 15 mmHg and 16 mmHg in the right and left eye, respectively. The general characteristics of the enrolled patients compared by gender are presented briefly in Table 1. Various ophthalmologic conditions have been identified in the selected sample, and the results are presented in Table 2.

**Figure 1:**
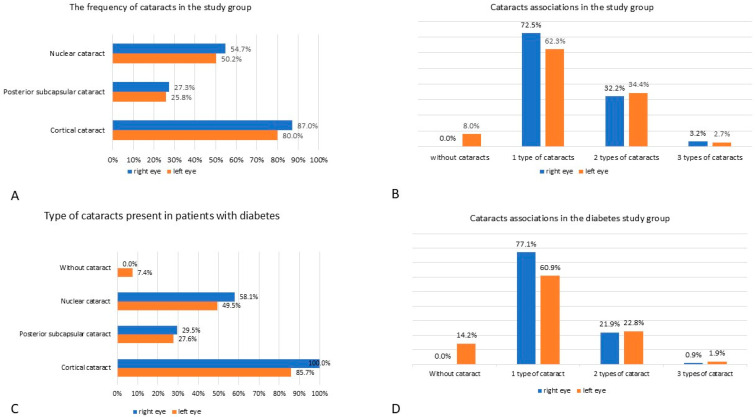
The frequencies of different types of cataracts in the studied patients. (**A**) the frequency of cataracts in the study group. (**B**) cataracts associations in the study group. (**C**) type of cataracts present in patients with diabetes. (**D**) cataracts associations in the diabetes study group.

92.5% (186/201) of the included patients presented with different types of cataracts in both eyes: 87.0% (175/201) and 80.0% (161/201) had CC in their right and left eye respectively; 27.3% (55/201) and 25.8% (52/201) had PSC; NS was found in 54.7% (110/201) individuals in the right eye and 50.2% (101/201) individuals in the left eye. These are presented in Figure 1A.

Of the 201 patients with cataracts, 52.2% of patients also presented with diabetes (7.6% (8/105) patients with T1D and 92.3% (97/105) patients with T2D), of which 52.4% were men. Patients with diabetes presenting with cataracts were significantly younger than non-diabetes patients with cataracts (66 years versus 77 years, *p* < 0.0001). The median duration of diabetes was 15 years. A total of 22.4% (45/201) were diagnosed with PND, and 84.8% presented different types of retinopathies: nonproliferative (mild 3.8%, moderate 8.6%, and severe 32.4%) and proliferative in 40%. Notably, 32.3% (65/201) of the patients were active smokers. The smoking habit was found to be similar in frequency regardless of diabetes presence. IOP values were similar in both eyes, and all studied patients showed no significant differences across diabetes or non-diabetes groups. Additionally, it has been shown that CC is significantly more commonly found in patients with diabetes (*p* < 0.0001). A summarization of the patients’ characteristics can be found in Table 3.

Another finding was that pseudophakia was less frequent in patients with diabetes—36.2% (38/105) compared to 56.2% (54/96) patients without diabetes, *p* = 0.004, in the right eye; 35.2% (37/105) patients with diabetes compared to 50.0% (48/96) patients without diabetes, *p* = 0.03, in the left eye. More than 62.3% of patients had one type of cataract present, unilateral or bilateral, and about 32.2% associated two types of cataracts at once, unilateral or bilateral. In contrast, only a small number of patients had three types of cataracts associated (Figure 1B).

In patients with cataracts and diabetes, 100% (105/105) patients presented CC in their right and 85.7% (90/105) in their left eye. PSC was present in 29.5% (31/105) patients in their right eye and 27.6% (29/105) patients in their left eye. NS affected the right eye of 58.1% (61/105) patients with diabetes and the left eye of 49.5% (52/105) patients (Figure 1C).

When assessing the group of patients with diabetes, the following data about cataract associations emerged: 77.1% (81/105) patients had 1 type of cataract in their right eye, 60.9% (64/105) patients in their left eye; 21.9% (23/105) patients associated two types of cataract in their right eye and 22.8% (24/105) in their left; only 0.9% (1/105) patients associated three types of cataract in their right eye, and 1.9% (2/105) patients in their left (Figure 1D).

In the present study, we further compared the presence and types of cataracts in patients with diabetes to those without. The results highlight significant differences in cataract prevalence and the distribution of cataract types between the two groups. It has been shown that CC is more frequently encountered in patients with diabetes (*p* < 0.0001 for the right eye and *p* = 0.03 for the left eye) and that patients with diabetes may have a higher rate of bilateral cataract involvement than patients without diabetes. Additionally, although statistical significance was not reached in the other types of cataracts, patients with diabetes had marginally higher prevalences of developing this condition (Table 4).

The model summary of random forest classification showed 25 trees, three features per split, 128 n (train), 33 n (validation), and 40 n (test), with 0.606 validation accuracy and 0.675 test accuracy. The confusion matrices indicate that the model struggled with classifying subtype 3 accurately, particularly with low precision for this class due to the low percentage of patients (Table 5).

**Figure 2:**
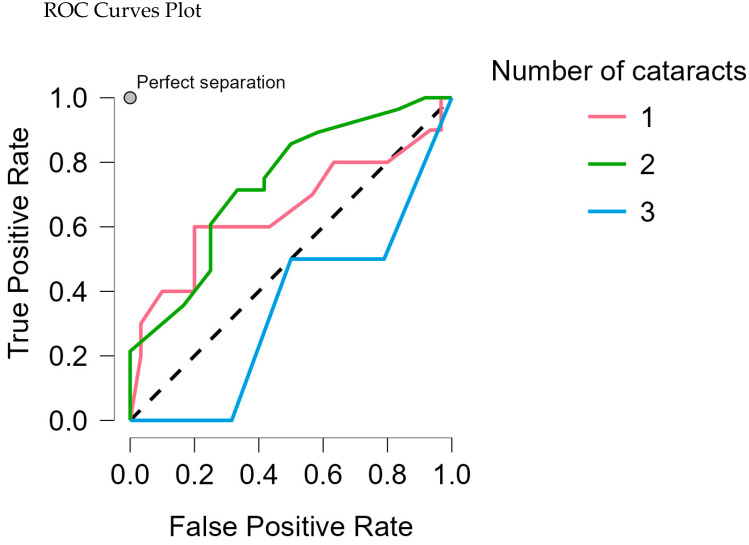
ROC curves plot each cataract association against all other classes.

In the Random Forest Classification model, the true positive rate for class 2 was 0.786, indicating that about 78% of true cases for this subtype were correctly identified. The false positive rate was 0.200 for class 1, meaning approximately 20% of non-cases were incorrectly predicted as having this subtype (Table 6, Figure 2).

**Figure 3:**
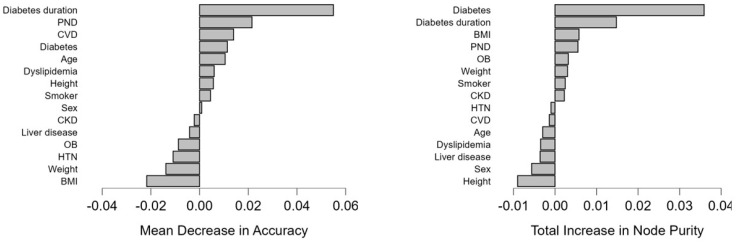
Graphical representation of the variable mean decrease and the variable total increase in node purity for the model.

Diabetes and diabetes duration had the highest mean decrease in accuracy (0.011, 0.055, respectively), the highest total increase in node purity (0.036, 0.015, respectively), and mean dropout loss (60.191, 73.329, respectively). This suggests that they are vital predictors of cataract subtype associations and effectively separate different cataract subtype associations. Also, the presence of PND showed a higher mean decrease in accuracy (0.022) with a moderate increase in node purity (0.006), suggesting a possible influence on the prediction model (Table 7, Figure 3). This indicates that diabetes patients will develop at least two types of cataracts during their diabetes progression; the longer the disease duration, the more likely it is to develop more cataracts.

## 4. Discussion

### 4.1. Findings and Their Interpretation

The present research aimed to evaluate the most frequent cataract subtypes in diabetic patients. Our study found that PSC and NC cataracts were marginally more common in patients with diabetes. Similarly, the Beaver Dam Eye Study also identified an association between diabetes and cataract formation [24]. The study took place over five years and comprised 3684 participants aged 43 and older. It showed an increased incidence and progression of PSC cataracts in diabetic patients. PSC cataract is known to impact a younger demographic diabetic group [25], and its development and progression are influenced by other risk factors besides diabetes, like ionising radiation, solar radiation, atopia, steroid use, hyperparathyroidism or other ophthalmologic pathologies such as myopia or retinitis [26]. These aspects emphasise the importance of a thorough ophthalmologic evaluation focused on cataracts, even in younger diabetic patients.

Similarly, the Blue Mountains Eye Study, population-based cross-sectional research, aimed to evaluate the relationship between NS, CC, and PSC cataracts in 3654 participants [27]. This study confirmed earlier findings about the detrimental effects of diabetes on the lens, demonstrating a statistically significant association between diabetes and cataracts. Additionally, the Visual Impairment Project assessed risk factors for cataract development in Australians and found that diabetes, when present for more than 5 years, was an independent risk factor for PSC cataracts [28,29].

In our study group, the prevalence of CC was significantly higher in patients with diabetes compared to those without diabetes, results which can be conflicting in the literature. While some studies indicate that CC occurrence is often independent of sugar level fluctuations, other studies continue to identify CC as one of the most common cataract subtypes among patients with diabetes, alongside PSC [15]. In this idea, an analysis of the Beaver Dam Eye Study examined the prevalence of cataract development in a population of 4926 adults, where diabetic patients were more prone to developing cortical lens opacities and had a higher rate of prior cataract surgery compared to those without diabetes [14,24,30,31]. Nevertheless, in our study, pseudophakia was encountered with a higher frequency in patients without diabetes, leading to the necessity of raising awareness regarding this impactful comorbidity and the need for an earlier diagnosis and treatment.

Another important observation was that in our study group, we noted that the median age at which patients with diabetes develop cataracts is significantly lower than that of individuals without diabetes (66 years vs. 77 years, respectively; Mann–Whitney Test, *p* < 0.001). Multiple clinical studies have demonstrated that cataracts develop more often and at an earlier age in individuals with diabetes compared to those without this condition [32,33,34]. Data from the Framingham study reveal that individuals with diabetes under the age of 65 have a three to four times higher prevalence of cataracts, while those over 65 have up to twice the prevalence compared to non-diabetic patients. This finding is supported by a study by Memon et al., which shows that among individuals under 40, 33.3% of people with diabetes develop cataracts compared to 16.4% of non-diabetics. For the 40–59 age group, the prevalence is 41% in people with diabetes versus 14.5% in non-diabetics. Similarly, in those over 60, 47% of people with diabetes develop cataracts compared to 16.4% of non-diabetics [11]. Becker et al. obtained similar results in a retrospective observational study involving 56,510 patients with diabetes [35]. The estimated incidence rates of cataracts were 20.4 per 1000 person-years (95% CI, 19.8–20.9) in patients with diabetes, compared to 10.8 per 1000 person-years (95% CI, 10.5–11.2) in the general population.

Age-related cataract is a condition that eventually affects both eyes equally, with the development of lens opacities that belong to all main cataract subtypes [36]. Nevertheless, there is a difference between these opacities that depends on the presence of certain diseases such as diabetes as well as factors such as glycemic control. It is essential that in patients with diabetes, we identify the early presence of cataracts as well as the subtype—since the majority of the patients had one type of cataracts, such that the patient can benefit from appropriate management that would allow him or her to have a favorable evolution and a better quality of life.

Our results also align with multiple studies that evaluate the association of cataract subtypes, especially among the elderly population [13,37]. It has been shown that the impact of cataracts on vision varies according to the localization of the opacity, leading to diverse symptoms in each of the aforementioned subtypes. When the opacity is centered in the lens (nuclear cataract), it tends to affect distal vision more than the proximal vision. Conversely, if the opacity appears as spokes (cortical cataracts), patients may experience glare and monocular diplopia [13]. When the opacity is situated just inside the posterior lens capsule (PSC), it can lead to considerable visual impairment as the opacities develop in the axial part of the lens. Also, depending on the cataract subtype, the progression rate varies, with PSC cataracts being known to have a faster progression rhythm than NC or CC [13].

Cataracts can significantly disrupt daily activities, and the extent of the consequences on the patient’s well-being can be self-reported using specific questionnaires such as the VF-14 [38]. Healthcare professionals worldwide are well aware of the importance of patients’ complaints. Therefore, recent guidelines consider symptomatic cataracts a surgical condition [39]. Still, when considering cataract surgery in diabetic patients, some precautionary measures should be considered, ranging from preoperative considerations to intra- and postoperative ones, as this surgery is known to increase the risk of both diabetic macular edema and diabetic retinopathy [40]. Cataract surgery should be considered not just in order to improve the patient’s quality of life but also their safety. This is highlighted by a study conducted by Meuleners et al., which found a 36% to 47% decrease in car crashes after cataract surgery in the first and second eye, respectively, among drivers older than 55 years [41].

In this context, where the timing of a diagnosis and treatment contributes immensely to a good prognosis of a patient with cataracts, it appears that technological developments and machine learning could have a key role in the improved management of these patients. Although results are still scarce, it has been shown that age has the strongest influence on the risk of NC and CC, alongside other factors such as an increased glucose level, data similar to the ones of our study, where the presence of diabetes and diabetes duration have been shown to have the highest importance for a successful classification [42]. Additionally, artificial intelligence (AI) could contribute to an early prediction of cataracts and to an efficient screening through automatic detection and grading, results that should be further explored [43,44].

Taking these aspects into consideration, in conjunction with our present results, we emphasize the importance of a thorough and timely evaluation of cataracts and their subtypes, with consecutive management that allows a favorable evolution and a better quality of life, especially among patients with diabetes that are generally individuals with multiple comorbidities [45]. Last but not least, besides a proper cataract evaluation and an optimal ophthalmologic therapeutic approach, managing risk factors involved in cataract development and progression remains of great importance. The main risk factors regarding cataract formation include elevated HbA1c levels and generally poor metabolic control. Besides the administration of glucose-lowering medications, lifestyle modifications, such as physical exercise and individualised dietary adjustments, remain of great importance among diabetic patients. Physical exercise and its intensity must be individualised according to each patient’s general health status, comprising a total of at least 150 min per week [46]. Dietary plans should also be individualised, with special attention on diets that affect gut health and dysbiosis, as gut microbiome status is known to be linked to inflammation, insulin resistance, and diabetes development [47]. All these management tools are essential in obtaining optimal glycemic control and lowering diabetes-related pathologies that are connected to hyperglycaemia, such as cataracts [48].

### 4.2. Strengths and Limitations of the Study

This present research lays out an extensive analysis of the most frequent cataract subtypes among patients with diabetes from Romania (Central Eastern Europe) that have been insufficiently evaluated regarding this pathology. Furthermore, as the patient’s enrolment in this study was made consecutively, in the order of their attendance to their prescheduled visits at the Emergency County Hospital “Pius Brinzeu” Timisoara, the final research group presented heterogeneous characteristics regarding gender, age, diabetes profile and presence of comorbidities, emphasizing the variety of patients that healthcare providers come across in day-to-day clinical practice. More so, the relatively large study population accurately reflects general and ocular health status among elderly patients on our side of the country. Additionally, the exploration of machine learning, in the context of a scarcity of results in the literature, could bring value to improving the diagnosis and management of cataracts in daily clinical practice.

Still, the present research has a few limitations. The consecutive enrolment of patients resulted in an elderly study population, yet, as cataracts are known to develop and progress even among younger diabetic patients, for future research, we acknowledge the importance of extending the evaluated population to a younger demographic group, regardless of the presence of diabetes [11,18,48]. Also, for future reference, multicenter research should be considered to expand and generalize present results. At the same time, the cross-sectional study design could be switched to a prospective one for a more accurate representation of cataracts and their interactions.

### 4.3. Relevance of the Findings

This study highlights the importance of an early and thorough ophthalmological evaluation of a vision-threatening disease—cataracts—and its subtypes in the general population, which is even more important for patients with diabetes. It is known that they are more prone to develop this debilitating condition. Furthermore, the present results emphasize the importance of screening patients with diabetes using technological developments such as machine learning prediction models or AI, with much-needed multidisciplinary participation to attain the best standard of care for each patient.

### 4.4. Future Perspectives

As mentioned above, future research refers to expanding the demographic group, including a younger evaluated population, while also considering a multicenter study protocol. Another perspective regards the evaluation of cataract and cataract subtypes in a prospective manner—rather than a cross-sectional design—to assess a possible time-dependent predominance and co-occurrence of different cataract subtypes. Furthermore, additional parameters could be included, like visual acuity, refractive errors, cataract surgery prevalence, and last but not least, questionnaires that asses patients’ quality of life before and after cataract surgery while exploring in more detail the benefits of using machine learning algorithms and AI, all of these in order to gain a better understanding on how each cataract subtype impacts patients’ daily activities and how to establish more efficiently the best therapeutic approach for these individuals.

## 5. Conclusions

While cataracts are a frequently encountered condition in the general population, especially among the elderly, patients with diabetes are more likely to have bilateral cataracts at a younger age, with a higher prevalence of CC—the most common subtype in patients with diabetes—but also PSC and NS. Patient management regarding vision-threatening cataracts should involve frequent ophthalmologic screening, especially among younger diabetic patients, and a subsequent medical and surgical treatment plan that is centered on the multiple ways diabetes impacts post-surgical vision outcomes. Additionally, machine learning could contribute to an early diagnosis of cataracts, with diabetes disease, diabetes duration, and PND being of the highest importance for a successful classification. These results could contribute to the improvement in screening and management of cataracts, especially in the vulnerable population of patients with diabetes, with further improved outcomes, including sight and increased quality of life.

## Figures and Tables

**Table 1:** The general characteristics of the enrolled patients compared by gender.

Parameter	Overall (*n* = 201)	Men (*n* = 98)	Women (*n* = 103)	*p*-Value *
Age ^a^ (years)	72 (63; 77)	69.0 (88.8)	74.0 (112.5)	0.003
Weight ^a^ (kg)	80 (72; 90)	89.0 (133.6)	75.0 (69.9)	<0.0001
Height ^a^ (cm)	170 (165; 180)	180.0 (146.1)	165.0 (58.0)	<0.0001
BMI ^a^	27.3 (25.7; 29.3)	27.4 (99.8)	27.2 (102.0)	0.7
Right IOP ^a^ (mmHg)	15 (13; 18)	15.0 (95.7)	15.0 (105.9)	0.2
Left IOP ^a^ (mmHg)	16 (14; 18)	15.0 (96.2)	16.0 (105.5)	0.2

BMI = body mass index; IOP = intraocular pressure; ^a^ Continuous variables are indicated by their median (interquartile range) or (average rank); * For comparison between genders with Mann–Whitney test.

**Table 2:** Additional ophthalmologic conditions encountered in the selected sample.

Ophtalmologic Condition	Frequencies
Anterior pole/cornea (zona zoster, herpetic keratitis, vortex keratopathy, cornea guttata, pterygium)	7.5% (15/201)
Glaucoma	17.9% (36/201)
Macular degeneration	13.9% (28/201)
Retinal vascular diseases	4.5% (9/201)
Retinal tear/detachment	8.0% (16/201)
Chorioretinal degenerative diseases	9.5% (19/201)
Inflammatory disease	2.0% (4/201)

**Table 3:** The general characteristics of enrolled patients compared by the presence of diabetes.

Parameter	Overall (*n* = 201)	Diabetes Patients	Non-Diabetes Patients	*p*-Value *
Male gender	48.8% (98/201)	52.4% (55/105)	44.8% (43/96)	0.2
Age ^a^ (years)	72 (63; 77)	66.0 (71.8)	77 (132.9)	<0.0001
Weight ^a^ (kg)	80 (72; 90)	80.0 (107.3)	79.5 (94.1)	0.1
Height ^a^ (cm)	170 (165; 180)	170.0 (98.0)	169.5 (104.2)	0.4
BMI ^a^	27.3 (25.7; 29.3)	28.2 (113.7)	26.8 (87.0)	0.001
Right IOP ^a^ (mmHg)	15 (13; 18)	15.0 (102.6)	15.0 (99.1)	0.6
Left IOP ^a^ (mmHg)	16 (14; 18)	16.0 (101.2)	15.0 (100.7)	0.9
Smoker ^b^	32.3% (65/201)	29.5% (31/105)	35.4% (34/96)	0.3
Dyslipidemia ^b^	71.6% (144/201)	66.6% (70/105)	77.1% (74/96)	0.1
HTN ^b^				<0.0001
None	7.0% (14/201)	0	14.5% (14/96)	
Grade 1	19.4% (39/201)	29.5% (31/105)	8.3% (8/96)	
Grade 2	45.3% (91/201)	45.7% (48/105)	44.8% (43/96)	
Grade 3	28.4% (57/201)	24.8% (26/105)	32.3% (31/96)	
CVD ^b^	50.7% (102/201)	58.0% (61/105)	39.0% (41/105)	0.02
CKD ^b^	27.9% (56/201)	35.2% (37/105)	19.8% (19/96)	0.01
Liver disease ^b^	12.9% (26/201)	15.2% (16/105)	10.4% (10/96)	0.3
Weight status ^b^				0.007
Normal-weight	17.9% (36/201)	16.2% (17/105)	19.8% (19/96)	
Overweight	63.2% (127/201)	55.2% (58/105)	71.8% (69/96)	
Grade 1	14.9% (30/201)	21.9% (23/105)	7.3% (7/96)	
Grade 2	2.5% (5/201)	3.8% (4/105)	1.0% (1/96)	
Grade 3	1.5% (3/201)	2.8% (3/105)	0	
Cataracts ^b^				
NS	57.7% (116/201)	63.8% (67/105)	51.0% (49/96)	0.06
CC	87.5% (176/201)	100% (105/105)	73.9% (71/96)	<0.0001
PSC	27.3% (55/201)	29.5% (31/105)	25% (24/96)	0.4

BMI = body mass index; IOP = intraocular pressure; HTN = arterial hypertension; CVD = cardiovascular disease; CKD = chronic kidney disease; NS = nuclear cataracts; CC = cortical cataracts; PSC = posterior subcapsular cataracts; ^a^ Continuous variables are indicated by their median (interquartile range) or (average rank); ^b^ Categorical variables are presented by the absolute frequency (percentage) and the number of individuals in the sample; * for comparison between groups.

**Table 4:** Comparison of Cataract Status and Types Between Patients with and without DM.

Variables	Patients with Diabetes (*n* = 105)	Patients Without Diabetes (*n* = 96)	*p*-Value *
Cataract type			
Cortical cataract—right eye	105 (100%)	70 (72.9%)	*p* < 0.0001 *
Cortical cataract—left eye	90 (85.7%)	71 (74.0%)	*p* = 0.03 *
Nuclear cataract—right eye	61 (58.1%)	49 (51.0%)	*p* = 0.3
Nuclear cataract—left eye	52 (49.5%)	49 (51.0%)	*p* = 0.8
Posterior subcapsular cataract—right eye	31 (29.5%)	24 (25.0%)	*p* = 0.4
Posterior subcapsular cataract—left eye	29 (27.6%)	23 (24.0%)	*p* = 0.5

* Chi-squared test, statistical significance threshold at *p* < 0.05.

**Table 5:** Confusion matrix and class proportions for random forest classification.

Confusion Matrix					Class Proportions				
		Observed				Data Set	Training Set	Validation Set	Test Set
		1	2	3					
Predicted	1	0.12	0.15	0	1	0.338	0.359	0.364	0.250
	2	0.12	0.55	0.05	2	0.602	0.563	0.636	0.700
	3	0	0	0	3	0.060	0.078	0.000	0.050

**Table 6:** Model performance metrics for predicting cataract associations.

	1	2	3	Average/Total
Support	10	28	2	40
Accuracy	0.725	0.675	0.950	0.783
Precision (Positive Predictive Value)	0.455	0.759	NaN	0.645
Recall (True Positive Rate)	0.500	0.786	0.000	0.675
False Positive Rate	0.200	0.583	0.000	0.261
False Discovery Rate	0.545	0.241	NaN	0.393
F1 Score	0.476	0.772	NaN	0.659
Matthews Correlation Coefficient	0.291	0.208	NaN	0.249
Area Under Curve (AUC)	0.662	0.676	0.151	0.496
Negative Predictive Value	0.828	0.455	0.950	0.744
True Negative Rate	0.800	0.417	1.000	0.739
False Negative Rate	0.500	0.214	1.000	0.571
False Omission Rate	0.172	0.545	0.050	0.256
Threat Score	0.294	1.100	0.000	0.465
Statistical Parity	0.275	0.725	0.000	1.000

Note. All metrics are calculated for every class against all other classes.

**Table 7:** Feature importance metrics for predicting cataract associations.

	Mean Decrease in Accuracy	Total Increase in Node Purity	Mean Dropout Loss
Diabetes	0.011	0.036	60.191
Diabetes duration	0.055	0.015	73.329
BMI	−0.022	0.006	60.693
PND	0.022	0.006	54.581
OB	−0.009	0.003	54.565
Weight	−0.014	0.003	60.594
Smoker	0.004	0.002	53.050
CKD	−0.002	0.002	52.643
HTN	−0.011	−9.776 × 10^−4^	61.512
CVD	0.014	−0.001	58.661
Age	0.010	−0.003	62.280
Dyslipidemia	0.006	−0.003	54.121
Liver disease	−0.004	−0.004	52.409
Sex	8.839 × 10^−4^	−0.006	56.207
Height	0.006	−0.009	62.773

Note. Mean dropout loss (defined as cross-entropy) is based on 50 permutations. BMI = body mass index; PND = diabetic polyneuropathy; OB = obesity; HTN = arterial hypertension; CVD = cardiovascular disease; CKD = chronic kidney disease.

## Data Availability

The data presented in this study are available on request from the corresponding author. The data are not publicly available due to local privacy and data protection regulations.
